# Rare pelvic peritoneal defect causing small bowel obstruction in a young female with virgin abdomen

**DOI:** 10.1093/jscr/rjad718

**Published:** 2024-01-10

**Authors:** Yunpeng (Jack) Deng, Emily Baker, Sumeet Toshniwal

**Affiliations:** General Surgical Department, Eastern Health, Box Hill, Victoria 3128, Australia; General Surgical Department, Eastern Health, Box Hill, Victoria 3128, Australia; General Surgical Department, Eastern Health, Box Hill, Victoria 3128, Australia

**Keywords:** small bowel obstruction, internal hernia, peritoneal defect, Pouch of Douglas

## Abstract

This case report discusses a 46-year-old female with no prior surgical history who presented with severe abdominal pain and generalized tenderness. She was found to have a small bowel obstruction secondary to internal hernia caused by a rare congenital pelvic peritoneal defect in the Pouch of Douglas. She required diagnostic laparoscopy and repair of the pelvic peritoneal defect. Congenital peritoneal defect is an extremely rare cause of small bowel obstruction but should remain a possible differential diagnosis in patients with virgin abdomen presenting with acute abdominal pain.

## Introduction

Congenital peritoneal defect in the Pouch of Douglas (POD) is a rare cause of internal hernia which can cause small bowel obstruction (SBO). Patients with a ‘virgin’ abdomen who presents with severe abdominal pain and generalized tenderness, who are suspected to have SBO, should warrant early surgical intervention in the form of laparoscopy or laparotomy. Prompt surgical intervention with reduction of the bowel within the internal hernia and repair of the peritoneal defect will be sufficient in most scenarios; however, delays in clinical judgment for surgery may precipitate ischemic and non-viable bowel, which requires bowel resection.

## Case report

A 46-year-old female with no previous surgical history presented to Emergency Department (ED) with 1 day history of sudden onset generalized abdominal pain and associated nausea and two episodes of vomiting. Her pain was described as sharp, severe, and radiates from the left to the right of her abdomen. She had a medical history of adult attention deficit/hyperactivity disorder (ADHD), anxiety, and Implanon (contraceptive implant). She was medicated with Ritalin for ADHD and drinks a few glasses of wine per week. On initial examination by an ED doctor, she had normal vital signs and her abdomen was soft, with generalized tenderness but no peritonism or guarding.

Initial blood tests showed negative B-HCG level for pregnancy, normal inflammatory markers, liver function test, and hemoglobin. Her urine dipstick test was also unremarkable.

**Figure 1:**
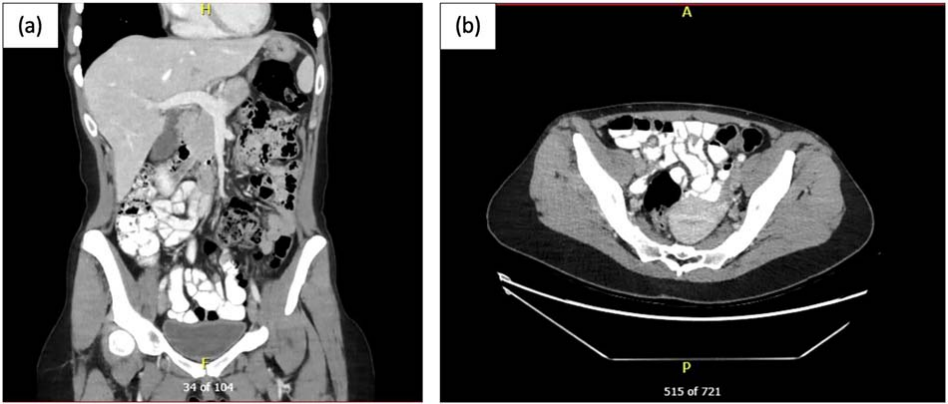
CT AP post oral and intravenous contrast; (a) coronal view and (b) axial view showing multiple mildly dilated small bowel loops, suspicious for developing SBO, with transition point in the right lower pelvis, where there is possible focal mural thickening, and no perforation; the appendix appeared normal.

Given the acuity of patient’s severe abdominal pain and generalized tenderness on examination, a computed tomography of the abdomen and pelvis (CT AP) was performed to assess for an acute surgical cause as shown in Fig. 1.

The CT AP demonstrated an evolving SBO which is consistent with patient’s clinical symptoms and presentation; however, the small bowel showed a maximal diameter of only 2.4 cm. General Surgery team was consulted promptly and instigated management involving insertion of a nasogastric tube (NGT) that was on free drainage and 4-hourly manual aspirations, bowel rest, indwelling catheter insertion for strict fluid balance, intravenous fluid resuscitation, and electrolyte optimization. In view of the patient’s pain and generalized tenderness on examination, an evolving SBO on CT AP in a patient with no previous abdominal surgery, a decision was made for urgent diagnostic laparoscopy.

**Figure 2:**
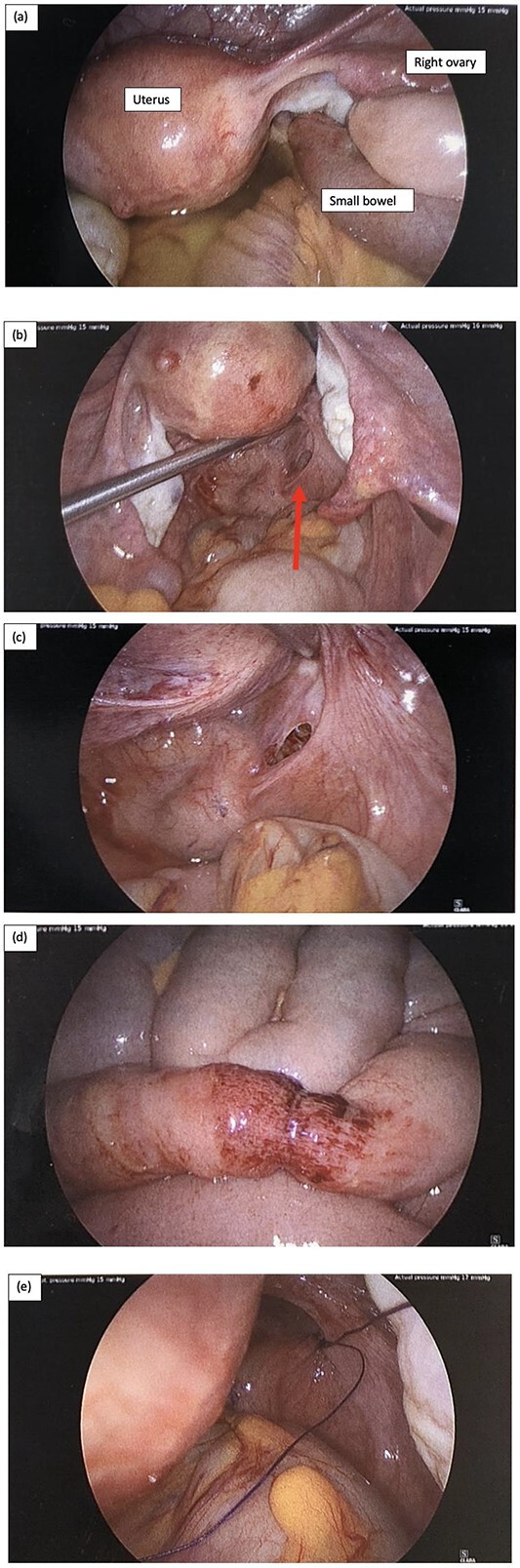
Intraoperative laparoscopy findings, and (a) key landmarks including the uterus and right ovary are identified, as well as a short segment of small bowel diving deep into the pelvis, and there is also moderate volume of serous free fluid; (b) serous fluid has been suctioned and there is small bowel herniating through a small peritoneal defect adjacent to the rectum on the right represented by the red arrow; (c) peritoneal defect in the POD; (d) the short segment of small bowel that was involved incarcerated in the hernia has a small area of bruising but is otherwise viable, and (e) the peritoneal defect was repaired with a single figure of 8 stich using 0 PDS suture.

Patient was operated on within 2.5 hours of surgical assessment. Laparoscopic surgery was performed with 12-mm Hassen optical infraumbilical port and 3 further 5 mm ports in the suprapubic, left iliac fossa and right iliac fossa regions. Intraoperative findings are demonstrated in Fig. 2.

There was a finding of a nubbin of small bowel herniating through a small peritoneal defect at the peritoneal reflection adjacent to the rectum on the right and moderate serous free fluid was identified (Fig. 2a–c). The small bowel was reduced easily with gentle traction using laparoscopy instruments and a single figure of 8 repair using 0 PDS suture was used to close the peritoneal defect (Fig. 2e). The short segment of small bowel which was incarcerated within the hernia was bruised as shown in Fig. 2d but viable. The small bowel was examined from duodenum to the terminal ileum and no other injuries were identified. The appendix was also mildly injected and removed laparoscopically. Patient was allowed sips of clear fluid postoperatively and NGT continued on free drainage and 4-hourly manual aspirations.

Patient had a quick and uneventful recovery after the operation. Day 1 postoperation, she was upgraded to free fluids, and NGT and IDC were subsequently removed. Patient was discharged on day 2 postoperation; she was clinically well with minimal abdominal pain; patient had good appetite and was upgraded to full ward diet. Abdominal examination was unremarkable.

Patient was reviewed in the outpatient clinic 2 weeks later in which she had a full recovery with no complications. The histopathology analysis of the appendix showed that it was within normal limits and no evidence of acute appendicitis.

## Discussion

Congenital pelvic peritoneal defect is an extremely rare cause of internal hernia and SBO [1]. Internal hernia attributes up to 5.8% of all causes of SBO [1, 2]. Internal hernias can be categorized as congenital or acquired, and the most prevalent forms of congenital internal hernia are para-duodenal (53%) and pericecal (13%), while supravesical and pelvic hernias make up ~6% of internal hernias [1, 2]. Predisposing risk factors for acquired internal hernia are mesenteric defect post bowel resection, iatrogenic creation of foramen post Roux-en-Y surgery, and trauma [1].

There are very few cases reported in the literature of congenital peritoneal defect in the POD causing an internal hernia and SBO. Suwa *et al.* (2013) [3] reported a very similar case involving a 28-year-old female with no prior surgery to have a peritoneal defect in the POD as the cause of SBO. The patient presented with symptoms of abdominal pain and vomiting, who later required laparotomy and repair of the peritoneal defect. The peritoneal defect was regarded as congenital given the patient had no prior pelvic surgery, pregnancy, childbirth, or trauma, which are viewed as risk factors for pelvic hernias [2]. There are only 4 other cases reported in the literature of POD peritoneal defects causing an internal hernia, written by Fiirgaard *et al.* (1988), Inoue *et al.* (2002), Bunni *et al.* (2011), and Hari *et al.* (2020) [4–7]. However, in the study by Hari *et al.*, the patient was a 33-year-old female who had cesarean section 4 years prior and Inoue *et al.* described an 80-year-old-female with previous hysterectomy and appendicectomy 30 years before.

Congenital peritoneal defects are not exclusive to the POD and appears to have the potential to affect any intra-abdominal region covered by the peritoneum. There have been 2 case reports of patients with peritoneal defect in the vesicouterine space as described by Odeh *et al.* (2023) and Mou *et al.* (2016), which also lead to internal hernia and SBO requiring surgical intervention [8, 9]. It was also discovered by Song in 2021 a congenital peritoneal defect formed by the liver and retroperitoneum causing SBO in a gentleman in his 40’s with no prior abdominal surgeries who required laparoscopy, reduction of hernia, and repair of peritoneal defect [10].

## Conclusion

Congenital peritoneal defect in the POD is an extremely rare cause of internal hernia which can be a transition point for mechanical SBO. It is often difficult for clinicians to make early diagnosis given its rarity and non-specific symptoms of abdominal pain, nausea, and vomiting. It should raise an alarm in patients presenting with SBO with no previous abdominal surgeries that a congenital peritoneal defect may be the cause. This warrants early surgical intervention such as diagnostic laparoscopy for definitive diagnosis and treatment.
